# Left ventricular diastolic function is strongly correlated with active emptying of the left atrium: a novel analysis using three-dimensional echocardiography

**DOI:** 10.1186/s12947-016-0085-0

**Published:** 2016-10-07

**Authors:** Johannes Scherr, Philip Jung, Tibor Schuster, Lars Pollmer, Gert Eisele, Franz Goss, Jens Schneider, Martin Halle

**Affiliations:** 1 Department of Prevention and Sports Medicine, Klinikum rechts der Isar, Technische Universitaet Muenchen, Georg-Brauchle-Ring 56, D-80992 Munich, Germany; 2 Medizinische Klinik und Poliklinik I, Klinikum der Universität München, Munich, Germany; 3 Department for Medical Statistics and Epidemiology, Klinikum rechts der Isar, Technische Universitaet Muenchen, Munich, Germany; 4 Heart Center “Alter Hof”, Munich, Germany; 5 Universitäts Herz-Zentrum Freiburg - Bad Krozingen, Klinik für Kardiologie und Angiologie II, Bad Krozingen, Germany; 6 Munich Heart Alliance, Munich, Germany

**Keywords:** Three-dimensional echocardiography, Left atrium, Left ventricular diastolic function

## Abstract

**Background:**

Increased left atrial (LA) dimensions are known to be a risk factor in predicting cardiovascular events and mortality and to be one key diagnostic tool to assess diastolic dysfunction. Currently, LA measurements are usually conducted using 2D-echocardiography, although there are well-known limitations. Real-time 3D-echocardiography is able to overcome these limitations, furthermore being a valid measurement tool compared to reference standards (e.g. cardiac magnetic resonance imaging).

We investigated LA function and volume and their association to left ventricular (LV) diastolic function, using newly designed and validated software for 3D-echocardiographic analysis. This software is the first to allow for a sophisticated analysis of both passive and active LA emptying.

**Methods:**

We analyzed 2D- and 3D-echocardiographic measurements of LA volume and function in 56 subjects and compared the results between patients with normal LV diastolic function (NDF) (*n* = 30, 52 ± 15 years, BMI 24.7 ± 2.6 kg/m^2^) and patients in which diastolic dysfunction (DDF) was suspected (*n* = 26, 65 ± 9 years, BMI 26.7 ± 3.7 kg/m^2^).

**Results:**

Volumes during LA active emptying were significantly smaller in DDF compared to NDF (active atrial stroke volume (ASV): 3.0 (0.1–4.5) vs. 5.5 (2.7–7.8) ml, *p* = 0.005; True-EF: 7.3(0.1–11.5) vs. 16.2 (8.1–25.4) %, *p* = 0.002). Furthermore, ASV showed a stronger association to E/e’_mean_ than all other measured LA volumes (β = − 0.35, *p* = 0.008). Neither total stroke LA volume, nor maximum or minimum LA volume differed significantly between the groups.

**Conclusions:**

Diastolic LV dysfunction results in a reduction in active LA emptying, which is more strongly associated with LV filling pressure than other previously investigated LA parameters.

## Background

Heart failure (HF) with preserved ejection fraction (EF) (HFpEF) significantly contributes to morbidity, mortality and health care costs in both the U.S. and Europe [1]. Currently the best non-invasive diagnostic strategies and criteria to characterize HFpEF have yet to be determined, largely because opinions addressing diagnostic strategy differ substantially between cardiac associations [2, 3].

The left atrium (LA) seems to play a pivotal role in the development of HFpEF, as LA size is strongly associated with left ventricular (LV) diastolic function and is an independent predictor of heart failure hospitalization in subjects with preserved ejection fraction and coronary heart disease [4, 5]. Suggested mechanisms of ventricular filling modulation are strongly related to the reservoir, conduit, and pump functions of the LA [6, 7].

As a potential tool in the measurement of LV diastolic function, LA volume is considered to reflect the cumulative effects of filling pressure over time in terms of atrial remodeling. This would be superior to the currently used technique of measuring left ventricular inflow, which only reflect the filling pressure at the time of measurement [3]. Therefore, LA volume seems to be a good surrogate parameter for cardiovascular risk, as well as a powerful predictor of cardiovascular outcomes [4, 8].

Left atrial size is clinically assessed by linear 2-dimensional (2D) echocardiographic (2DE) measurements in 2- and 4-chamber views, which provides a fairly good estimation of the true dimension [9]. Although this measurement has been accepted as having sufficient clinical feasibility and reliability, in recent years 3-dimensional (3D) echocardiography (3DE) assessment has become increasingly available.

The 3D-echocardiographic measurements for chamber quantifications have shown high correlations with the gold standard of cardiac magnetic resonance imaging (cMRI) and computed tomography (CT) [10–12]. In contrast to cMRI and CT, which is costly both in terms of time and money, 3DE is a relatively fast and economical bedside method to assess LA size and function [13]. Furthermore, other LA parameters such as the left atrial function – generated by both the passive early filling of the left ventricle (LV) caused by the movement of the valvular plane and the active atrial contraction – were rarely analyzed until the introduction of 3DE.

In a recent study, it was shown that a decreased contribution of active left atrial emptying to ventricular filling during diastole was strongly predictive of adverse cardiac events and death [14].

Therefore, we performed the first evaluation of passive and active left atrial function and size using newly designed, already validated software for 3D-echocardiographic analysis and its correlation with left ventricular diastolic function [11, 12].

## Methods

### Study population

Sixty consecutive, randomly assigned subjects (14 women, 46 men) who underwent echocardiography with a commercially available ultrasound system (iE33, Philips Healthcare, Hamburg, Germany) were recruited from 1) the out-patient clinic for Prevention and Sports Medicine, Klinikum rechts der Isar, Technische Universitaet Muenchen or 2) the Division of Cardiology, Department of Medicine, Medizinische Klinik und Poliklinik, Ludwig-Maximilians-Universität, Campus Innenstadt.

The study protocol was approved by the university’s ethical board (Klinikum rechts der Isar der Technischen Universitat Munchen) and the investigation conforms to the principles outlined in the Declaration of Helsinki. All participants gave written informed consent.

Inclusion criteria were age ≥18 years and written informed consent. Exclusion criteria were significant cardiac valvular disease (at least moderate (2nd degree) mitral or aortic regurgitation or mitral stenosis), cardiac arrhythmias or conduction abnormalities (e.g. (intermittent) atrial fibrillation or flutter, left or right bundle branch block (QRS duration >120 ms), pacemaker rhythm, frequent premature beats), previous cardiac surgery, reduced left ventricular ejection fraction (EF <55 %), previous transcoronary ablation of septal hypertrophy (TASH), acute coronary syndrome (ACS) within four weeks or myocardial infarction within 2 months, or severe pulmonary hypertension with clinical relevant right ventricular impairment.

The participants were divided into the following groups: (1) a control group consisting of subjects with an E/e’_mean_ < 8; and (2) subjects with an E/e’_mean_ ≥ 8 and also maximal LA volume (LAV_max_) ≥ 34 mL*m^−2^ and therefore meeting criteria for diastolic LV dysfunction according to current guidelines using 2DE [2, 3]. Study design aimed to include 30 subjects in each group. Four participants were excluded because of very poor image quality (as recommended by the current guidelines [15]). In the group with normal diastolic function (control group), mainly leisure time athletes (all performance category ranging from hobby sportspeople to former elite athletes, all being free of any cardiovascular diseases) were examined who presented within the Department of Prevention and Sports Medicine for primary prevention purposes. In the group with evidence of diastolic LV dysfunction, mainly patients with heart failure with preserved ejection fraction (HFpEF) were included (recruited both in the Department of Prevention and Sports Medicine and Division of Cardiology).

Hypertension was defined as previously described [16]. Hyperlipidemia was defined as a total fasting cholesterol level of more than 6.21 mmol/L or use of lipid-lowering medication.

### Echocardiography

Transthoracic echocardiographic investigations (standard 2D parasternal short- and long-axis images and apical 2-, 3- and 4-chamber views, and 3-dimensional echocardiography) were conducted during end-expiratory apnea in a left lateral decubitus position by experienced echocardiographers in accordance to current recommendations [17]. Echocardiographic images were collected at all sites and analyses were performed at the core laboratory at the Department of Prevention and Sports Medicine. Indexes of LA volumes for body surface area were calculated as previously described with a variation of the formula from DuBois [18]:

$$ \mathrm{B}\mathrm{S}\mathrm{A}{\left[{\mathrm{m}}^2\left]=0.007184\times \mathrm{Height}\right[\mathrm{cm}\left]{}^{0.725}\times \mathrm{Weight}\right[\mathrm{kg}\right]}^{0.425}. $$

#### 2D echocardiography (2DE)

For 2D-echocardiography, a transthoracic broadband S5-1 transducer (frequency transmitted 1.7 MHz, received 3.4 MHz, Philips Medical Imaging, Hamburg, Germany) was used.

2D atrial size was assessed with the biplane method of discs (modified Simpson’s rule) and the area-length method from apical two- and four-chamber views as recommended by the American Society of Echocardiography [9]. The maximal length of the LA was measured at ventricular end-systole in an apical-four chamber view. Maximal left atrial volume (LAV_max_) was measured at the ventricle end-systole just before opening of the mitral valve. Minimal left atrial volume (LAV_min_) was measured at the end-diastole on ECG just before closure of the mitral valve [9].

Peak velocities of trans-mitral inflow during early filling (E), atrial contraction (A), and the deceleration time of the E-wave velocity (DT) were measured in apical four-chamber view using pulsed Doppler echocardiography with positioning of the Doppler sample volume perpendicular to the flow jet at the tips of the mitral valve leaflets; E/A ratio was subsequently calculated. The early diastolic mitral annular velocity (e’) was measured at the septal side as well as the free LV wall of the mitral annulus using tissue Doppler imaging on the longitudinal axis in the apical 4-chamber view. E/e’_med_ and E/e’_lat_ as well as E/e’_mean_ (calculated as E/mean(e’_med_ and e’_lat_)) were subsequently calculated.

Diastolic function was graded as “normal” when E/e’_mean_ was <8 and “at least suggestive of diastolic dysfunction” when E/e’_mean_ was ≥8 and LAV_max_ was ≥34 mL*m^−2^ [2, 3, 19]. The latter one with evidence of diastolic dysfunction was subdivided into the three stages of diastolic dysfunction in accordance to the classification of Khouri [20]. Also in the current guidelines, consistency between at least two indices is required to make the diagnosis of a diastolic dysfunction [15]. We chose E/e’_mean_ and LAV_max_ because these indices seem to be feasible and reproducible [15]. Furthermore, LAV_max_ represents a marker of chronicity of elevated LA pressure [15]. Additionally, E/e’_mean_ ratio seems to be less age dependent than other indices and therefore also suitable to compare groups with different mean ages [15]. An E/e’_mean_ ratio <8 usually indicates normal LV filling pressure [15].

#### Real-time 3D-echocardiography (RT3DE)

For each patient, RT3DE was performed directly after 2DE with a 3 to 1 MHz transthoracic matrix array transducer (X3-1) in the harmonic mode from an apical window to acquire full-volume 3D images in accordance to the current recommendations [17]. To encompass the entire left heart into the real-time 3-dimensional echocardiographic data set, a full volume up to 92° × 84° scan was acquired from seven R-wave-triggered sub-volumes during an end-expiratory breath-hold. The depth and angle of the ultrasound scan sector were adjusted to the minimal level still encompassing the entire left ventricle and atrium. The temporal resolution of the data sets ranged from 25 to 34 ms.

Analysis of systolic function of the left ventricle (3D-EF_LV_) and left ventricular volumes (LV stroke volume [LV-SV]) was performed as previously described [21].

**Fig. 1:**
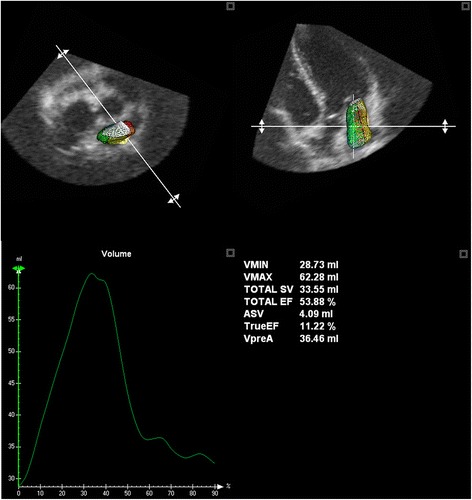
3D image analysis (4D LA Function, TomTec Imaging Systems) depicting the performed measurements

3D LA volume (3D-LAV) was analyzed with RT3DE software (4D LA Function, TomTec Imaging Systems, Munich, Germany) as previously described (see Fig. 1) [11, 12]. 3-dimensionally measured maximum left atrial volume (LAV_max3D_) was assessed at ventricular end systole right before opening of the mitral valve by semiautomatic tracing of the LA endocardial surface. Minimal 3-dimensionally measured volume (LAV_min3D_) was measured at ventricular end-diastole just after closure of the mitral valve. Total stroke volume of the left atrium (Total SV) was calculated as the difference between the minimum and maximum left atrial volumes.

After manual setting of five to seven tracing points in each view, the endocardial border was automatically delineated, and the LAV was obtained automatically throughout the heart cycle, resulting in LA volume-time curves. LA appendages and the confluence of the pulmonary veins were excluded from the tracing. Manual corrections were made to modify the automatic tracings in some subjects where necessary (inaccuracy of endocardial automated detection). To differentiate between early diastolic filling (E = LA passive emptying) and atrial contraction (A = LA active emptying) a marker (preA) was set at the moment of the second opening component of the biphasic opening movement of the mitral valve. Corresponding volume was defined as pre-atrial contraction volume (V_preA_). For LA, atrial stroke volume (ASV) was calculated as LAV_preA_ − LAV_min3D_. Total-EF was calculated as (LAV_max3D_ − LAV_min3D_)/LAV_max3D_ × 100 %. True-EF was calculated as ASV/LAV_max3D_ × 100 %. Left atrial conduit volume (LA-CV) was calculated as followed: LA-CV = LV-SV − Total SV. Additionally, LA-CV was expressed as percentage of LV stroke volume (LA-CV_rel_) to present the magnitude of LA-CV on LV systolic function.

3D LA size and function were interpreted blinded to the 2D measurements of diastolic function.

### Statistics

Data analysis was performed using PASW Statistics 22.0 (SPSS Inc., Chicago IL, USA). For quantitative data, the mean, standard deviation and range or if more appropriate (non-normally distributed data) the median and interquartile range (IQR: 25th/75th percentile) were reported for descriptive purpose. Assumption of normal distribution of data was verified by using descriptive methods (skewness, outliers and distribution plots) and inferential statistics (Shapiro–Wilk test).

Non-normally distributed main outcome parameters were logarithmically transformed prior to parametric data analysis. Thus, relative effects of potential explanatory variables were modeled. Back-transformation was performed using simple exponential functions. Thus back-transformation of regression coefficients gives an estimate for the median relative change of outcome measure by a one-unit increment of the corresponding explanatory variable.

For correlation analyses of left ventricular inflow parameters and parameters of left atrial function, Spearman correlation coefficients (ρ) were calculated. Agreements between 2D- and 3D-methods were assessed by the Bland–Altman analysis.

We performed a receiver operating characteristics (ROC) analysis with participants meeting criteria for diastolic LV dysfunction (E/e’_mean_ ≥ 8 and also maximal LA volume (LAV_max_) ≥ 34 mL*m^−2^) as event of interest and various quantities of the 3D assessment as potential predictors. Areas under the ROC curve (AUCs) and corresponding 95 % confidence intervals are presented. For the most important measures (True-EF, Total-EF, and ASV) the ROC curves are shown.

For analysis of the interobserver variability, measurements were performed by two blinded observers. To assess intraobserver variability, measurements were repeated 2 weeks later by an observer blinded to the previous measurements. Inter- and intraobserver variabilities were calculated as the difference between the two measurements in terms of the percentage of their mean.

A *p*-value <0.05 was considered to indicate statistical significance. Testing was performed two-sided.

## Results

### Participants’ characteristics

Baseline characteristics and 2-dimensional echocardiographic data are presented in Table 1.

**Table 1:** Baseline characteristics of participants

	Normal diastolic function (E/e’_mean_ < 8) *n* = 30	With evidence of diastolic LV dysfunction (E/e’_mean_ ≥ 8 & LAV_max_ ≥ 34 mL/m^2^) *n* = 26	*p*-value
Anthropometry			
Women [%]	4 (13 %)	10 (38 %)	0.07
Age [yrs]	52.3 ± 15.1	64.8 ± 9.3	0.001
Weight [kg]	76.5 ± 9.7	78.3 ± 12.8	0.63
Height [m]	1.76 ± 0.07	1.71 ± 0.08	0.02
Body mass index [kg/m^2^]	24.7 ± 2.6	26.7 ± 3.7	0.01
Fat Free Mass [kg]	63.0 ± 9.5	57.2 ± 6.8	0.09
Body surface area [m^2^]	1.92 ± 0.14	1.90 ± 0.18	0.55
Cardiovascular Risk Factors			
Hypercholesterolemia (total cholesterol ≥6.21 mmol/L)	5 (17 %)	7 (27 %)	0.61
RR_sys_/RR_dia_	128 ± 16/83 ± 10	144 ± 22/85 ± 9	0.01/0.25
Hypertension (RR_sys_ > 140 mmHg or RR_dia_ > 90 mmHg)	9 (30 %)	16 (62 %)	0.07
Echocardiography			
Heart rate [bpm]	59 ± 11	68 ± 10	0.001
3D-EF_LV_ [%]	59.7 ± 5.2	60.6 ± 12.0	0.14
LV mass [g/m^2^]	86.6 ± 16.0	96.9 ± 30.4	0.35
E [cm/s]	68.4 ± 18.1	79.6 ± 16.4	0.02
Left ventricle internal diameter, diastole [mm]	47 ± 6	39 ± 11	0.002
Posterior wall thickness, diastole [mm]	11 ± 2	13 ± 3	<0.001
Septum wall thickness, diastole [mm]	11 ± 2	14 ± 3	<0.001
A [cm/s]	60.6 ± 14.5	79.2 ± 20.0	0.001
E/A ratio	1.20 ± 0.44	1.05 ± 0.35	0.10
Dct [ms]	218 ± 8	262 ± 6	0.004
e’_lat_ [cm/s]	14.0 ± 3.6	8.9 ± 2.7	<0.001
E/e’_lat_	5.01 ± 1.12	9.9 ± 4.6	<0.001
e’_med_ [cm/s]	9.3 ± 2.8	6.4 ± 1.5	0.001
E/e’_med_	7.42 ± 1.57	13.01 ± 3.46	<0.001
E/e’ _mean_	5.55 ± 1.33	11.30 ± 2.92	<0.001
E/e’ _mean_ [range]	2.9–7.9	8.0–20.0	<0.001

Mean ± SD or median (IQR)

(*RR*_*sys*_ */RR*_*dia*_ systolic and diastolic blood pressure, *E* early diastolic filling peak velocity, *A* peak velocity during atrial contraction, *Dct* Deceleration time of the E-wave velocity, *e’*_*lat*_ early diastolic mitral annular velocity free LV wall, *e’*_*med*_ early diastolic mitral annular velocity septal)

In the group with evidence of diastolic dysfunction (*n* = 26), 14 (54 %) had impaired relaxation (stage I diastolic dysfunction), 7 (27 %) had pseudonormal function (stage II diastolic dysfunction) and 5 (19 %) had a reversible restriction (stage III diastolic dysfunction).

### 3-dimensional echocardiographic parameters

**Fig. 2:**
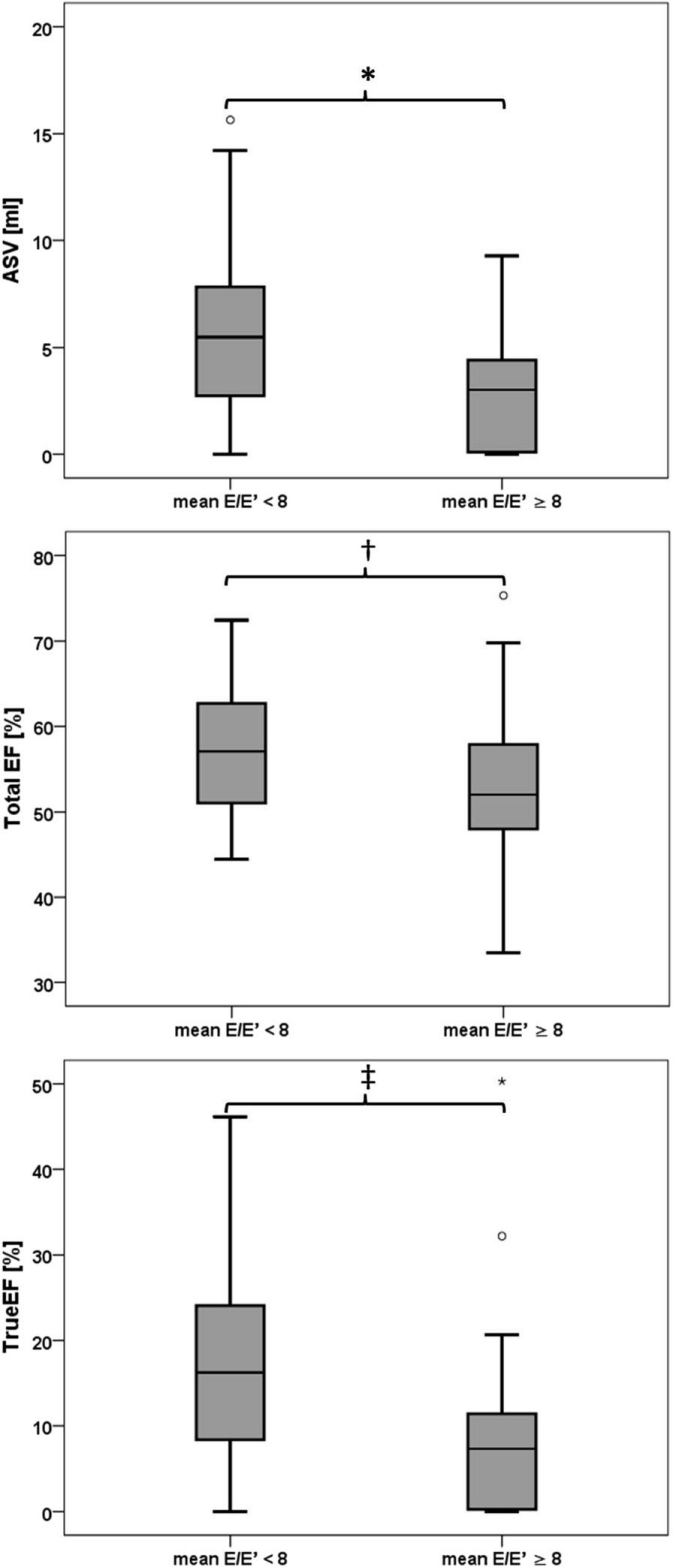
LA volumes and function in the investigated groups. * indicating *p* = 0.005, †indicating *p* = 0.09, ‡ indicating *p* = 0.002

No significant between-group differences were observed in left atrial dimensions (both minimal and maximal volume and total stroke volume) when measured with 2DE and 3DE. However, there were significant differences in volumes caused by active left atrial emptying, like ASV and true atrial ejection fraction (see Table 2 and Fig. 2). In these investigations, the group with suspected diastolic dysfunction had decreased atrial stroke volume (median [IQR]: 3.0 [0.1–4.5] ml vs. 5.5 [2.7–7.8] ml, *p* = 0.005), also when corrected for BSA and BMI, respectively. Furthermore, the true ejection fraction was significantly lower in the group with diastolic impairment (7.3 vs. 16.2 %, *p* = 0.002).

**Table 2:** 3D-echocardiographic characteristics (volumetric and functional) of the left atrium

	Normal diastolic function (E/e’_mean_ < 8) *n* = 30	With evidence of diastolic LV dysfunction (E/e’_mean_ ≥ 8 & LAV_max_ ≥ 34 mL/m^2^) *n* = 26	*p*-value
2DE			
LAV_Simpson_ (ml)	65.4 ± 20.2	69.8 ± 30.0	0.71
LAV_area-length method_ (ml)	55.3 ± 21.3	56.2 ± 10.1	0.27
3DE			
LAV_max3D_ (ml)	54.5 ± 13.2	57.1 ± 21.3	0.87
LAV_max3D_/BSA (ml/m^2^)	28.5 ± 7.2	30.3 ± 12.6	0.88
LAV_max3D_/BMI (ml × m^2^ × kg^−1^)	2.2 ± 0.5	2.2 ± 1.2	0.21
LAV_min3D_ (ml)	23.6 ± 7.4	28.4 ± 15.8	0.38
LAV_min3D_/BSA (ml/m^2^)	12.3 ± 4.0	15.1 ± 8.5	0.44
LAV_min3D_/BMI (ml × m^2^ × kg^−1^)	0.96 ± 0.31	1.10 ± 0.71	0.85
Total SV (ml)	30.7 (26.7–34.8)	27.1 (22.1–32.3)	0.13
Total SV/BSA (ml/m^2^)	15.4 (13.3–18.5)	14.1 (12.0–16.6)	0.16
Total SV/BMI (ml × m^2^ × kg-1)	1.24 (1.02–1.44)	1.03 (0.86–1.17)	0.009
ASV (ml)	5.5 (2.7–7.8)	3.0 (0.1–4.5)	0.005
ASV/BSA (ml/m^2^)	2.8 (1.3–4.3)	1.5 (0.0–2.4)	0.007
ASV/BMI (ml × m^2^ × kg-1)	0.21 (0.11–0.35)	0.10 (0.00–0.16)	0.002
V_preA_ (ml)	34.5 (27.3–42.2)	38.1 (28.0–51.4)	0.31
Total-EF (%)	57.0 ± 7.3	52.2 ± 11.9	0.09
True-EF (%)	16.2 (8.1–25.4)	7.3 (0.1–11.5)	0.002
LA-CV (ml)	31.6 (18.7–44.0)	29.9 (17.0–32.9)	0.27
LA-CV_rel_ (%)	52.3 ± 15.2	44.9 ± 17.9	0.11

Mean ± SD or median (IQR)

(*LAV* left atrial volume, *LAV*_*max3D*_ 3d-measured maximal left atrial volume, *LAV*_*min3D*_ 3d-measured minimal left atrial volume, *SV* stroke volume, *V*_*preA*_ pre-atrial contraction volume, *ASV* atrial stroke volume, *LA-CV* left atrial conduit volume, *LA-CV*_*rel*_ LA-CV expressed as percentage of LV stroke volume, *BSA* body surface area, *BMI* body mass index)

These significant associations between E/e’_mean_ and LA volumes were also supported in univariate and multivariate linear regression models. In the correlation analysis there were highly significant inverse associations between E/e’_mean_ and parameters of active left atrial emptying (correlation coefficients: ASV: *ρ* = −0.472; True EF *ρ* = −0.488, all *p* < 0.001). In contrast, total left atrial function (represented by Total SV and Total-EF) was not different between the groups (all *p* > 0.05). There were no significant associations between 2-dimensionally measured (such as A or a’ wave) and 3-dimensionally assessed (e.g. ASV) parameters of active LA emptying (all *p* > 0.05).

**Fig. 3:**
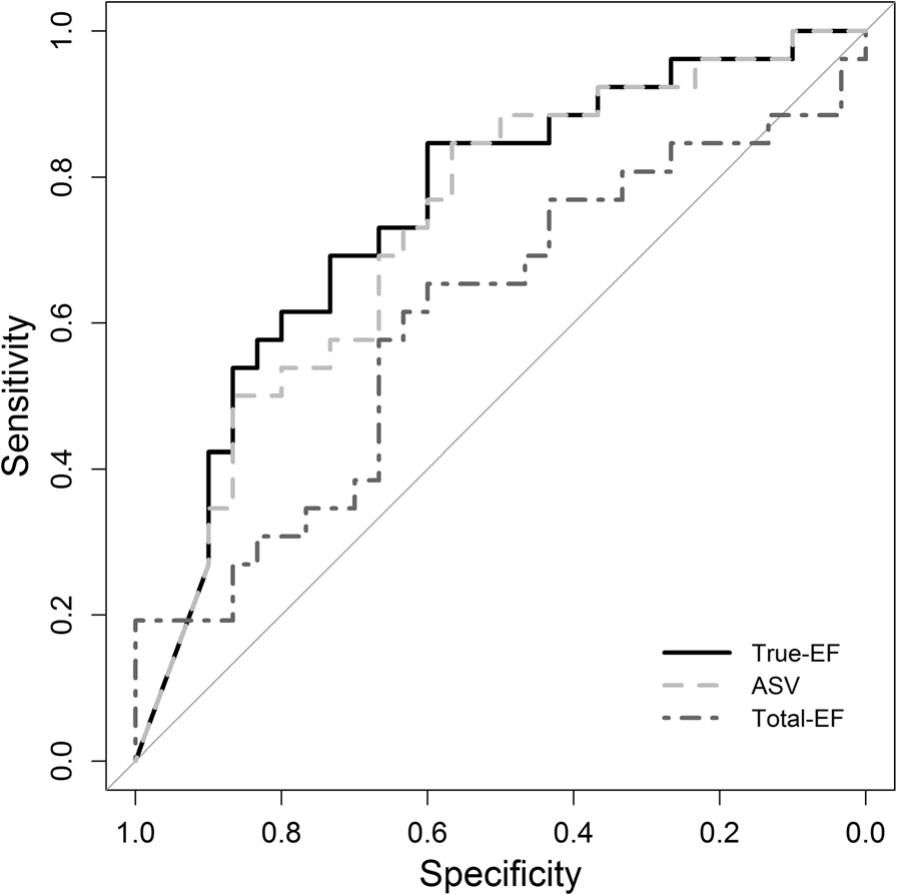
ROC curves for True-EF, ASV and Total-EF with respect to participants meeting criteria for diastolic LV dysfunction (E/e’_mean_ ≥ 8 and also maximal LA volume (LAV_max_) ≥ 34 mL*m^−2^) as event of interest

In ROC analyses, True-EF showed the highest AUC (0.753 (0.623–0.883)), followed by ASV (0.731 (0.598–0.865)) and Total-EF (0.605 (0.452–0.758)). These AUCs were not significantly different between the examined parameters (*p*-value ranging from 0.114 to 0.409). ROC curves are presented in Fig. 3.

In regression analyses, total SV (β = −0.024, *p* = 0.862) and LAV_max3D_ (β = 0.125, *p* = 0.357) showed weak associations to E/e’_mean,_ whereas LAV_min3D_ showed a modest association (β = 0.196, *p* = 0.148). ASV showed the strongest association (β = −0.421, *p* = 0.001). Also after adjustment for age, blood pressure, heart rate and LV mass, active atrial emptying (ASV) remained strongly associated with E/e’_mean_ in a linear regression model including all LA volumes (see Table 3).

**Table 3:** Linear regression of left atrial volumes with E/e’_mean_

	Unadjusted			Adjusted^a^		
	B (SE)	β	*p*-value	B (SE)	β	*p*-value
ASV	−0.41 (0.13)	−0.40	0.002	−0.35 (0.13)	−0.35	0.008
Total SV	−0.03 (0.05)	−0.08	0.55	−0.07 (0.05)	−0.19	0.19
LAV_min3D_	0.05 (0.047)	0.17	0.20	−0.02 (0.05)	−0.08	0.64

Values represent parameter estimates (B), standard errors (SE), and standardized parameter estimates (β)

^a^Adjusted for age, heart rate, hypertension and left ventricular mass

### Reproducibility

The correlation coefficients ranged from 0.90 (ASV) to 0.99 (LAV_min3D_ and LAV_max3D_) between measurements (performed by one observer, intra-observer variability) and from 0.90 (ASV) to 0.99 (LAV_min3D_) between observers (inter-observer variability). Cronbach’s Alpha ranged from 0.90 (ASV) to 0.99 (LAV_min3D_).

### Agreement of 2D- and 3D- determined left atrial volumes

**Fig. 4:**
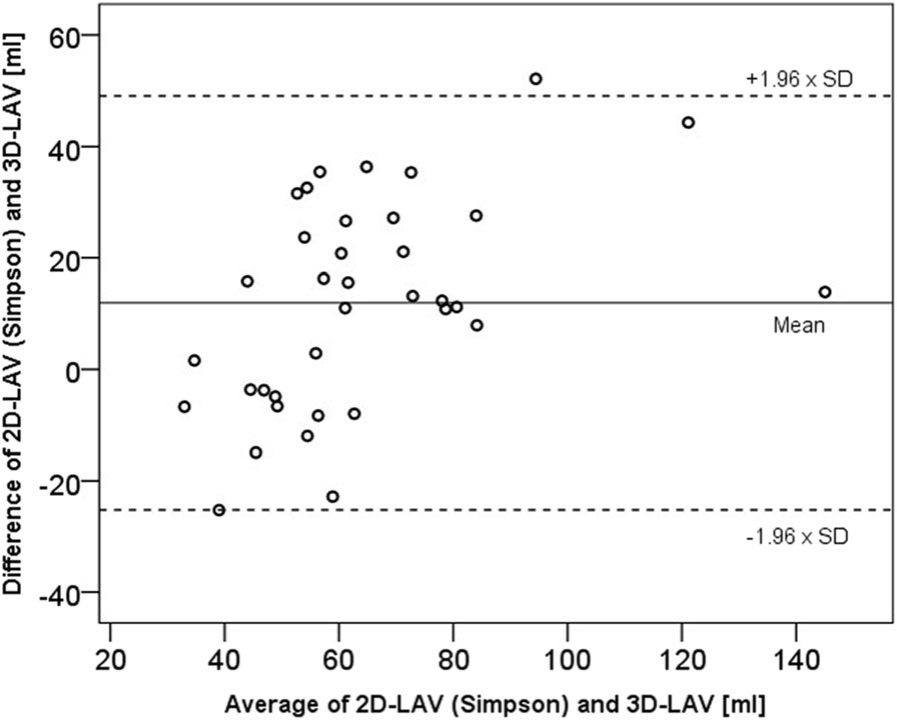
Bland–Altman plot comparing 2- and 3-dimensionally measured left atrial volumes

Agreement of 2-dimensionally and 3-dimensionally measured left atrial volumes are presented within Bland-Altman correlation (see Fig. 4). The left atrial volume measured 2-dimensionally using the Simpson method seems to overestimate the volume compared to the 3-dimensionally measurement. In contrast, LA volume analyzed with the area-length technique resulted in smaller sizes when compared to 3D measurements.

## Discussion

Our study is the first to analyze both active and passive LA emptying components with regard to diastolic function.

We were able to detect differences between a group with normal diastolic function compared to a group with participants representing with diastolic dysfunction with regard to active LA emptying. In contrast, all other parameters of LA volume showed no significant differences. Therefore, it can be assumed that using 3DE with special focus on active LA emptying might result in earlier and more sensitive diagnosis of diastolic dysfunction compared to conventional approaches.

### New parameters and left ventricular diastolic function

In a recent study, Russo et al. observed that minimum LA volume was closer correlated to LV diastolic function than maximum LA volume [22]. This is in accordance with our results. In the multivariate regression analysis, we also found LAV_min3D_ to better correlate with LV diastolic function than LAV_max3D_. However, the newly investigated parameters of LA function (True-EF and ASV) showed even stronger associations and seem therefore to be a more sensitive instrument to measure small but meaningful alterations of diastolic function. One reason for this reduction during active LA emptying might be the decreasing compliance of the left ventricle with progressive filling at end-diastole in patients with impaired diastolic LV function [3].

Regarding another three-dimensionally assessed parameter (relative LA conduit volume) which was prior linked to diastolic dysfunction [23], we were not able to demonstrate an association between increased LA-CV_rel_ and impaired diastolic LV function. However, the populations between our study and the cohort of Nappo et al. differ significantly (e.g. LV-EF: 60.1 ± 9.3 % vs. 37.1 ± 11.3 %) and LV stroke volume and EF play a decisive role in the calculation of LA-CV_rel_ and determination of impaired diastolic LV function [15], the results of these two studies cannot be compared. Therefore, further studies are needed with larger numbers of participants to be able to calculate reference values which can be generalized.

In another recent study on 2-dimensional evaluation of LA volumes and function, Teo et al. observed that in subjects in the initial stages of diastolic dysfunction, the active emptying volume is increased compared to subjects with normal diastolic function [24]. However, as the grade of LV diastolic dysfunction increases, this compensatory mechanism reduces and is eventually lost as mechanical atrial dysfunction sets in, resulting in a lower total LA emptying volume. Similar results were observed by Prioli et al., while investigating left atrial volumes and function with 2D-echocardiography and left ventricular catheterization [7]. This is consistent with the current study in which nearly half of the participants had higher stages of diastolic dysfunction; however, we were able to observe decreases in active LA emptying even at the very early stages of diastolic dysfunction. The reason behind this might be due to the fact that the analyses of the LA volumes in both of the aforementioned studies were conducted with conventional 2D methods, which are known to be less precise then CMR imaging or 3D echo [12]. Furthermore, in the study of Prioli et al., left atrial volume and function were estimated based on 2D-measured ventricular filling volumes and mitral flow patterns.

Similar results to those of Teo and Prioli were demonstrated in a study of Murata et al., who were able to show in cases of impaired relaxation, an initial increase in active LA emptying followed by a decrease of active LA emptying in the stages of pseudonormalisation and restriction (and vice versa regarding passive LA emptying) [25].

However, in studies assessing left atrial volumes 3-dimensionally, the software application tool (QLAB, Philips Medical System, Andover, Massachusetts) used was originally designed for analysis of LV volumes. Therefore, the analysis of these studies might be less precise compared to the software we used and which was specifically designed for volumetric analysis of the left atrium [26].

### Clinical implications of new LA parameters

Studies investigating the impact of left atrium as a prognostic marker concentrate mainly on maximum LA volume [6, 27, 28]. This parameter can be assessed easily with 2D-echocardiography, and therefore there is a large amount of data available.

However, newer studies suggest that other measures of left atrium dimensions and function (e.g. minimum LA volume or ejection fraction) have higher prognostic impact and a closer correlation to left ventricular diastolic function than maximum LA volume [5, 14, 22, 29]. Especially LA function has attracted scientific attention recently. Epidemiologically, left atrial function seems to play an important role as a risk factor in all-cause mortality. In particular, LA True-EF seems to be superior to LAVI with respect to cardiovascular death but also all-cause mortality even after adjustment for traditional cardiovascular risk factors and LV parameters [29].

Therefore, future studies investigating the impact of left atrial volumes and function should focus on novel and easily accessible LA markers such as total left atrial function and active left atrial emptying. These markers can be determined validly with the new method including the software used in the current study and correlate well with conventional markers of diastolic function.

#### Limitations and strengths

It can certainly be argued that our study included a relatively small number of participants and data should be validated by larger patient cohorts. However, the sample was heterogeneous (both randomly selected healthy subjects referred to the out-patient clinic and seriously ill patients consulted in by cardiologists in a University Hospital were included) and therefore representative. Therefore, the findings of our study can be generalized.

Secondly, we did not perform direct measurements of LV end-diastolic pressure because this requires invasive procedures. However, we used Doppler-derived E/e’ ratio as a surrogate parameter of LV end-diastolic pressure. This method is routinely used in clinical settings and studies and has been clinically validated [30, 31]. Additionally, we refrained from strain or speckle LA analyses, which are parameters investigated in the context of diastolic function most recently [32, 33]. However, these investigations would go beyond the scope of the current paper and might reduce the clarity. Therefore, this might be a topic of future analyses.

Furthermore, recording of the total duration of the diastole was not possible due to the modality of acquisition which required a record of seven subsamples to attain a sufficient resolution. Therefore, LAV_min_ could not be measured with complete assuredness. However, there are two studies demonstrating that the measurement of minimum LA volume assessed by the new software correlated excellently with the gold standard measurements (cardiac magnetic resonance imaging and computed tomography) [11, 12]. Especially in the study of Mor-Avi et al. cMRI and 3D-echocardiography showed identical results regarding LAV_min_ (bias: 0 ml, *r* = 0.88) [12]. In the study of Rohner et al., LA_min_ measured by RT3DE was even lower than measured by CT [11]. Therefore, we are confident that the data for LA_min_ measured with RT3DE are valid. Furthermore, this limitation would be relevant for both the NDF and DDF groups, leaving the results based on group differences unbiased in this aspect. Nevertheless, future studies should examine whether a significant difference between the left atrial volumes analyzed in loops covering the whole diastole also exists.

However, there are several strengths of the current study. First of all, state-of-the-art techniques (e.g. 3D-echocardiography and tissue Doppler imaging) were used. The 3-dimensional examination procedure of left cardiac dimensions and function has been demonstrated to be superior to 2-dimensional investigations [12, 21].

Another strength of our study is the use of a novel, yet validated software tool which was specifically designed for detailed assessment of both left atrial volumes and dimensions. This software allows sophisticated analyses of both passive and active emptying of the left atrium, which allowed for the new results of the present study.

Lastly, our study was conducted with a multi-centre setting and therefore has greater general applicability than single-centre studies.

## Conclusion

Diastolic dysfunction causes a reduction in active left atrial emptying which is more strongly associated to left ventricular filling pressure than maximum or minimum left atrial volume.
